# Population health AI researchers’ perceptions of the public portrayal of AI: A pilot study

**DOI:** 10.1177/0963662520965490

**Published:** 2020-10-21

**Authors:** Gabrielle Samuel, Heilien Diedericks, Gemma Derrick

**Affiliations:** King’s College London, UK; University of Lancaster, UK

**Keywords:** AI, artificial intelligence, digital data, expectations, health, health technology, hype, interviews, media, newspaper, qualitative research

## Abstract

This article reports how 18 UK and Canadian population health artificial intelligence researchers in Higher Education Institutions perceive the use of artificial intelligence systems in their research, and how this compares with their perceptions about the media portrayal of artificial intelligence systems. This is triangulated with a small scoping analysis of how UK and Canadian news articles portray artificial intelligence systems associated with health research and care. Interviewees had concerns about what they perceived as sensationalist reporting of artificial intelligence systems – a finding reflected in the media analysis. In line with Pickersgill’s concept of ‘epistemic modesty’, they considered artificial intelligence systems better perceived as non-exceptionalist methodological tools that were uncertain and unexciting. Adopting ‘epistemic modesty’ was sometimes hindered by stakeholders to whom the research is disseminated, who may be less interested in hearing about the uncertainties of scientific practice, having implications on both research and policy.

## 1. Introduction

Scholars in the field of the sociology of expectations have long shown how news reporting of innovative health technologies is over-emphasised through ‘breakthrough narratives’ (Brown, 2003; Fortun, 2008; Hilgartner, 2015; Petersen, 2018; Samuel and Kitzinger, 2013), and how this hype is not simply a by-product of innovation, but rather constitutes an innovation process itself: by envisaging futures in the present, it creates a positive vision of the technology, which acts performatively by securing funding in the present (Lehoux et al., 2017; Samuel and Farsides, 2017; Van Lente, 2012). The consequences of these performative effects have been explored in terms of both stakeholders and the public (Hilgartner, 2015; Samuel et al., 2017; Samuel and Kitzinger, 2013), as well as on how clinicians and researchers manage such expectations in their day-to-day practices (Gardner et al., 2015; Will, 2010). Some of this work has pointed to a disconnect between sensationalised public depictions of innovative technologies, and researchers’ more sober interpretations (see, for example, Beaulieu, 2002; Dumit, 2004). Pickersgill (2016) describes this as researchers’ ‘epistemic modesty’ (Will, 2010) whereby researchers admit the uncertainty, ambiguity and opacity of their field of study, and juxtapose this with what they see as overly optimistic media representations. As Pickersgill describes, this ‘epistemic modesty’ underscores a general reflexive awareness of the limits of scientific knowledge. Collins (1997, 1999) explains that this disconnect emerges because ‘distance leads to enchantment’ (Collins, 1997): those close to science (researchers working on the science projects) are often aware of the uncertainties of the methods they use, but because scientists tend to ‘black box’ areas of controversy and uncertainty, ‘those distant from the research front [other researchers, policymakers, funders, the public], and thus not exposed to the art and craft of scientific practice, get a view of science relatively free of doubts and uncertainties’ (p. 165). As such, explains MacKenzie (1998), the perceived uncertainty about matters such as the reliability, safety or predictability of a technology is higher for those closer to knowledge production. The further removed from science one becomes, the less uncertain the research appears, leaving an ‘alien science’ (Collins 1999; Hedgecoe, 2006), in which a habitable ‘space’ emerges for socio-technical futuristic expectations to proliferate (Brown and Michael, 2003; Hedgecoe, 2006).

In the health domain, artificial intelligence (AI) systems are an example of an innovative health technology. AI is a branch of computer science involving the development of a range of heterogeneous computer algorithms to accomplish tasks traditionally associated with human intelligence, such as the ability to learn and solve problems (Tang et al., 2018). The general aim of AI research is to use computer algorithms to analyse their environment through data, uncover relevant information from the data, and make predictions about the data in order to take actions to achieve specific goals (He et al., 2019). In the health and medical domain, machine learning is probably the most commonly used AI system. In comparison to a general AI system, which is intended to perform most activities that humans can perform, machine learning constitutes ‘narrow AI’, and can perform only one or a few specific tasks. It includes, for example, deep learning, neural networks and natural language processing (Ho, 2019). Machine learning systems have been used to help in the development of diagnostic aids for, for example, eye disease, heart disease, stroke, Alzheimer’s Disease (Loh, 2018; Tran et al., 2019). They are also being used to improve medical imagining, where standardised images (x-rays, pathology slides, photographic images, etc) provide relatively uncomplex datasets for AI-based visual pattern recognition. Here, much work has explored the utility of AI-based systems in the prediction and diagnosis of various cancers, such as skin cancer and breast cancer. Alongside this, AI systems have been incorporated into robotic surgical devices; used for the detection of genetic disorders (Gurovich et al., 2019); and tested as a predictor of responses to certain medications (Loh, 2018). In population health research, researchers are exploring how AI systems can help predict epidemics and disease outbreaks (Bengtsson et al., 2015; Harris et al., 2017; Naghavi et al., 2010), as well as control current pandemics.^1^ They are also exploring how AI systems can help individuals with various health and mental health states through the use of ‘digital phenotyping’, for example, by using AI systems as a predictor for suicide attempts and depression (Birk and Samuel, 2020; Blasimme and Vayena, 2019).

Sensationalist discourses have often accompanied the reporting of AI research in a series of AI-hype bubbles that have repeatedly peaked and troughed over the past 70 years (Bory, 2019; Chuan et al., 2019; Elish and Boyd, 2018; Fast and Horvitz, 2016; Krijgsman, 2018; Laï et al., 2020; Natale and Ballatore, 2017). Such discourses closely link to two competing imaginaries of, on one hand, pessimism, with concerns around dystopian surveillance, and on the other hand, with utopian views of AI systems spurring innovation and acting as a powerful tool to address various societal ills (Boyd and Crawford, 2012; Brennen et al., 2018; Elish and Boyd, 2018; Fast and Horvitz, 2016; Krijgsman, 2018). While media discourses of AI have long been explored, to date, little is known about how the media specifically portray AI in the context of health research, or how health researchers navigate this.

The aim of this pilot paper is to draw on work studying promissory discourses and the sociology of expectations to explore how population health researchers working with AI systems in Higher Education Institutions (HEIs) navigate their work with relation to the public presentation of AI systems. The aim is to also triangulate these findings with a small scoping analysis of how the news media portray AI specifically in the context of health research. Our study focuses on the United Kingdom and Canada, with the latter from English-speaking regions only. We conducted 18 interviews with UK and English-speaking Canadian researchers, and a small pilot analysis of the way in which UK and English-speaking Canadian newspaper articles portray the use of AI systems for population health research.

## 2. Methods

### Rationale of study

Our interview study initially aimed to explore how HEI UK and Canadian AI population health researchers made ethics decisions about their research. We found that interviewees wanted to reflect critically on the public portrayal of AI systems and how this related to their own views of the technology. While rich data around ethics were produced (*forthcoming*), the focus on perceptions about the public portrayals of AI systems with respect to interviewees’ own research demanded further attention. Furthermore, given the context in which interviewees situated their own research, we wanted to triangulate their perceptions of the way in which AI systems were portrayed in the media with a scoping analysis of how UK and Canadian news articles actually portrayed AI systems specifically in the health research arena.

### Interviews

#### Recruitment

To identify UK researchers, a bibliometric search for UK publications reporting on health-related research using AI methodologies was conducted as per methods described previously (Samuel et al., 2019). Data were manually checked and cleaned by using web searches to check each data point (a specific author) for expertise and experience in the field of AI-associated population health research (*n* = 244 cleaned to *n* = 58). Researchers were invited for interview; 10 researchers agreed to participate. For Canadian researchers, bibliometric sampling was not required because the Canadian Institute of Health Research (CIHR) provided an initial list of relevant researchers, which was used for further snowball sampling. Fifteen researchers were invited for interview; eight agreed to participate. The higher Canadian response rate could be related to the recruitment method. We note that our interviewees are self-selected which may produce data biases. However, it was not our desire to achieve a representative sample of interviewees, rather, for this exploratory pilot project, we were interested in purposively sampling researchers with experience and expertise in AI population health research.

#### Demographics

The sample was mainly male (*n* = 14/18, reflecting the reported heavy bias in the field of machine learning AI; Leavy, 2018). Interviewees were from a range of seniority levels (8 Professors, research chairs or heads of research teams; 7 associate/assistant Professors or lectures/senior lecturers; 2 research fellows/associates; 1 PhD); from 14 different universities (9 UK, 5 Canadian); working with a range of data (clinical, health survey, user sensor); and from a range of disciplines, including computer science, informatics, data science, epidemiology, public health and statistics. Some interviewees identified themselves as straddling across two disciplines: *n* = 8 positioned themselves as computer scientists, *n* = 8 positioned themselves as population health, statistics and/or epidemiology experts, and *n* = 6 positioned themselves as data scientists and/or informaticians.

#### Data collection and analysis

Interviews were conducted in 2019, were face-to-face, over the telephone or through Skype, and were audio-recorded and semi-structured. The interview schedule was broad, exploring interviewees’ views regarding the ethical issues surrounding AI health research; their own experiences around this; as well as their views and experiences of decision-making around ethical approval processes. We note that during these interviews, the public portrayal of AI systems and its implications emerged early on as a key focus of interviewees’ narratives.

Analysis of interview data was approached inductively using two inter-linked rounds (Strauss, 1987). Initially, interview transcripts were carefully read and re-read for relevant ideas and themes, and combined with the extensive memo-making taken directly after interviews. Second, interview transcripts were analysed line-by-line using NVivo software. For this article, the emergent conceptual category of relevance was related to interviewees’ perceptions of public portrayals of AI, and it was this theme that was coded for. For the purposes of this article, and given the small sample size, all disciplines were treated equally in the analysis and no distinctions are made between UK and Canadian interviewees. Where differences between UK and Canadian interviewees are present, these are noted in the findings. The study received ethics clearance from King’s College Research Ethics Committee (MRM-18/19-10499).

### Media analysis

#### Data collection

Headlines and lead paragraphs of UK national newspapers, and Canadian newspapers with readerships over 100,000 in 2015 (https://en.wikipedia.org/wiki/Newspaper_circulation), were searched for between 1 July 2018 and 2019 using LexisNexis (a comprehensive, online news database containing news articles from around the world). Search terms included ‘AI’ or ‘artificial intelligence’ or ‘machine learning’ or ‘neural networks’. Search results were filtered into two independent sub-groups using the LexisNexis sub-field filtering system, including (a) ‘Health and Medicine’ and (b) ‘Science and Technology’: sub-set ‘medical science’, ‘biochemistry’, ‘biology’, ‘behaviour and cognition’, ‘emerging technology’. While incorporating other sub-groups, for example, those related to business and employment were considered; our aim was to conduct a small analysis particularly focused on the reporting of AI systems in health research to act as a triangulation of interview findings. After duplicates were removed and only articles focusing on AI in health research/care were included, 11 Canadian articles and 79 UK articles remained (*n* = 90). News articles’ word length ranged from 30–1400 words in the UK sample, and 30–1200 words in the Canadian sample.

#### Analysis

A text-only content and discourse analysis were conducted, informed by the methodological approaches of Murdoch and Caulfield (2018) and Nerlich and Halliday (2007). Each article was also read in detail to ensure understanding of its context. Coding categories for content analysis included newspaper, topic of focus and how balanced the article was (positive, negative, neutral). In terms of the latter, each article was analysed for the presence of benefits and concerns regarding AI. Articles noting benefits of the technology but omitting or only briefly mentioning contrasting views (e.g. possible harms or technical challenges associated with its implementation) were coded ‘positive’. Similarly, articles noting harms or challenges of the technology, but omitting or only briefly mentioning a contrasting view, were coded as ‘negative’. Articles were coded as ‘balanced’ if positive and negative aspects of AI were detailed in equal measure, or if a conclusive stance could not be determined.

Discourse analysis was informed by the work of Norman Fairclough, which involves three levels of analysis: a textual analysis providing a description of what is said and how it is said in the text; second, a closer reading establishing whether the data contained any of the coding categories or themes identified in the content analysis; and a final level of explanatory analysis identifying discursive elements in the data (Fairclough, 2013). Discourse analysis was performed on the headlines and lead paragraphs because these are the crucial components in communicating the intended information to readers. Research suggests that more than 40% of news readers do not read further than the news headline (API, 2014; CXL, 2018) and 59% of news links shared have never been clicked upon – that is, presumably only the headline has been read (Gabielkov et al., 2016). Other research suggests that both offline and online news headlines act as ‘relevance optimisers’, created to optimise the story’s relevance (Blom and Hansen, 2015; Dor, 2003; Scacco and Muddiman, 2016; Van Dijk, 1998) and that readers of off/online news content scan more and read less (Holmqvis et al., 2013; Van Dijk, 1988).

#### Limitations

Interview sample sizes do not allow generalisations or contrasts between different demographic data (e.g. the United Kingdom/Canada; computer scientist/statistician using ‘off-the-shelf’ AI software vs developing software). The small sample size was also a limitation for the news analysis, especially given the sample’s scarcity of Canadian newspaper articles, which hinders generalisations being made about the findings. Furthermore, we omitted French-speaking newspapers and interviewees from our analysis and so cannot draw conclusions about news content disseminated to French-speaking Canada (and in addition, the views of French-speaking researchers for our interviewee study). While the media analysis was appropriate for our aims to act as a triangulation of our interview findings, further research comprehensively exploring news media portrayals of AI across all news categories is needed.

## 3. Findings

### Interviews with AI researchers

#### AI is ‘hype’, in the public arena

During nearly all UK interviews (*n* = 9/10), and some Canadian interviews (*n* = 4/8), participants reflected upon, or were incredibly keen to discuss, how the notion of ‘AI’, as well as the implications attached to its use, were often simplistically promoted and exaggerated in public portrayals of the technology when compared to the actual capabilities of the discipline: ‘there is a culture of reporting artificial intelligence as though . . . it’s from the films rather than what it actually is’ (Interviewee 10). The possibilities attached to AI, or sensationalist ‘hype’, were perceived in two polar-opposite forms. First, some participants spoke about how the media enthused and over-exaggerated the potential societal good that this new innovative AI would bring: ‘I think there’s also a lot of enthusiasm or hype around this area and I think it’s easy to get carried away [in terms of the capabilities of AI]’ (Interviewee 8). Second, media narratives were perceived to revolve around discourses of a new ‘threat’, imminent harm and/or danger to society. This was commonly referred to as AI eventually ‘replace[ing] doctors’ (Interviewee 9) and leading to ‘robots telling you what to do’: ‘I think there is a concern which the threat kind of pedals quite a lot about, you know, could AI invade your life, and you’ll have a robot telling you what to do’ (Interviewee 7).

Interviewees pointed to a range of factors they perceived perpetuated this hype. Reminiscent of ‘alien science’ (Collins 1999; Hedgecoe, 2006), some described the incredible complexity of AI, reflecting how even those close to the research, such as clinicians and other AI researchers, were not always able fully to understand the capabilities and limitations of the technology. This lack of understanding led to confusion, and ultimately provided a habitable space in which AI could be hyped simply because of the difficulty and complexity in questioning the legitimacy of the hype (Hedgecoe, 2006):

AI is a complex topic [which can lead to hype]. Even many of the people involved in the research, so some medical doctors . . . as well as sometimes the AI researchers themselves who might come from a more theoretical background, they are not aware of perhaps technical limitations of the algorithm . . . so . . . there is a degree of confusion. (Interviewee 6)

Others described how the inclusivity and heterogeneity of the AI term, and the fact it encapsulates a multitude of methods, perpetuates AI as a constantly shifting definition of ‘the stuff that computers can’t do yet’. This was perceived to place the technology in the realms of imaginary science fiction and abounding future expectations and sensationalism:

when I was trained, we didn’t have spelling checkers, but it was given as an example of something that AI might do in the future. Now it’s so common we wouldn’t even [call it] AI, it’s just completely trivial. I think the boundary of what we consider AI is possibly moving, and it tends to be in this stuff that computers can’t yet do but is in this nice imaginary science fiction type of area. (Interviewee 5)

Interviewees also spoke about the different actors who had a role to play in expectation generation. In line with previous literature (Bory, 2019), Interviewee 10 used the example of Google DeepMind’s *Alpha Go* – the first computer programme to beat a professional human player at the board game *Go* – to highlight industry’s role in promoting hype. Widespread media coverage of the game heralded the software a ‘breakthrough’, though for this interviewee, the capabilities of this ‘limited system’ had been over-hyped:

the scientific reporting of that [the game] then came across as Google have created the best artificial intelligence. [But] it’s only a very limited system that’s able to do a specific task in a way that we haven’t thought of doing it before. (Interviewee 10)

Similar to other areas of innovative health technologies (see, for example, Pickersgill, 2016), interviewees also discussed the role of AI researchers in contributing to hype. Interviewee 18 was unimpressed with computer science’s role in hype generation:

Compsci [computer science] is almost sometimes like ‘we are going to replace you, and we have got solutions that you will become irrelevant’, And maybe that is harsh . . . they are very bravado and very disruptive like we are disrupting, AI is a disruptive technology and we are going to revolutionise medicine like it’s never been revolutionised.

Furthermore, using Cambridge Analytica as an example, Interviewee 7 was concerned that researchers were branding themselves as conducting ‘AI research’ because the excitement around the field would provide more funding, in spite of the fact that sometimes they were not actually doing AI research. This was an interesting example for this interviewee to choose given the wide-ranging issues that emerged from this scandal, though this does not negate the point this interviewee wished to emphasise. That is, that companies, and perhaps also researchers, associate themselves with conducting AI research because, in this interviewees’ perception, this AI branding leads to more attention and investment: ‘they [Cambridge Analytica] could have done that [their research] but not called themselves an AI company, they only called themselves an AI company because that looks good on the branding’.

#### Criticisms of hype: AI is just a non-exceptionalist research tool

Interviewees’ contrasted their own experiences of AI with their perceptions of its public portrayal. AI was not viewed as a new technology by interviewees, nor that ‘exciting’ (Interviewee 1). As Interviewee 13 explained, ‘machine learning [doesn’t] represent a whole new scary domain here. I think it’s just a bit of a step forward in a continuum researchers have always been on’. Interviewees described how we are still at the point where very little is understood about the technology, even by AI researchers themselves. As such, it was very unlikely, explained Interviewee 7, that ‘robots would be taking over the world’ because so much was uncertain about the capabilities of AI, in whatever capacity AI was defined. This was echoed by Interviewee 11, ‘I think although there’s a lot of hype about AI as you know, a lot of AI is generally not that useful and not that good. Maybe when it gets better it will be scarier but . . .’. Similarly, Interviewee 9 explained, AI will not be replacing doctors anytime soon, and was ‘not a threat’ to society or to healthcare; *they can*’*t replace all the doctors . . . no* [*they are not*], *not at the moment* . . ..

In fact, Interviewee 3 explained, AI was really just a rebranding exercise of maths and statistics – a maturing and mainstreaming of these fields around the use of big data. As Interviewee 15 remarked, ‘I haven’t labelled anything I’ve done as AI, just machine learning, which just means like statistical methods, as far as I know. Like more flexible statistical methods’. Using the example of an AI cancer diagnostic system, Interviewee 10 explained, that AI systems were not ‘all-knowing’, but rather suffered the usual issues and uncertainty of any technology using big data, such as false positives and false negatives, and of key importance was understanding how to interpret the information the AI software provided to clinicians:

a cancer diagnostic system, for example . . . actually there are false positives, there are false negatives . . . and so I guess it’s around understanding what that means . . . and being able to act properly, interpret the information that comes from them . . ..

Nearly all (*n* = 16/18) interviewees adopted this non-exceptionalist view of AI, perceiving the technology as just another research ‘*tool*’ with which to analyse big data (Interviewee 16); and analogous to other methodological tools such as predictive algorithms and (bio)statistics. As Interviewee 4 explained, *the way I would use AI in my research, it*’*s just another methodology to try and get an answer out of the data set*. For one interviewee, the non-exceptionalist view of AI was not just related to the AI ‘tool’ but also to the new-found collaborations with computer science disciplines:

over the last 2500 years we have [formed collaborations with other disciplines] every 100 or 200 years . . . We have worked with physiologists and lab tech. . . . [computer science] is a bit different, but is it different? (Interviewee 18)

The analogy of AI as a ‘tool’ also stretched into perceptions on how AI should be implemented at a societal level. In response to public portrayals of AI taking away clinicians’ jobs, Interviewee 8 explained that AI systems would merely act as a tool by removing the burden of monotonous tasks, freeing up space for such professionals to look at what was defined as the ‘harder issues’. They remarked,

I think what you’ll actually find is machine learning AI applications get used to remove some of the burdens from healthcare professionals on the easy and simple tasks that are amenable to automation . . . I think . . . it [will] free up time of healthcare professionals to look at the harder cases, the harder issues.

#### Perceptions about the effect of AI hype

Alongside being critical of AI hype, our interviewees were also acutely aware of its likely influence on stakeholders, including other researchers, health professionals, patients and the public, both in terms of individuals placing too much trust in AI, or by being overly fearful of it. At the research level, Interviewee 6 was concerned about validity-related implications of AI hype, and, in particular, that AI-associated hype distracted researchers from thinking through scientific issues around notions of correlation and causation. Using a ‘them and us’ narrative, this interviewee explained,

everyone knows correlation isn’t causation in the scientific world. But now the words for that are different. So instead of causation you’re thinking about root cause drivers [an AI term] . . . and instead of correlation you think about association, or the predictive model can predict . . . It is actually the same problem, but it is worded differently . . . and people tend to forget . . ..

Also at this research level, and also reflecting the ‘them and us’ narrative, other interviewees were concerned that public representations of AI portrayed the technology as ‘cool’, which attracted researchers to incorporate the methodology into their research practices, sometimes even inappropriately or unnecessarily. Interviewee 12 distanced themselves from this sub-par work, which they viewed was caused by other researchers who were reflecting less rigorously on their methodological approaches:

it’s still a little bit of, ‘this is cool, let’s run the algorithm and see what happens ..[..].. Do we understand it? It doesn’t matter, it’s complicated and it works’ . . .[..].. [but] you have to still go through this process, to kind of vet that the instrument that you have is understandable and more or less behaves how you think it will behave.

At the societal level, on one hand, the enthusiastic reporting of AI was viewed as a contributing factor to an ‘alien science’, and the over-trust clinicians placed in AI systems: ‘doctors tend to believe in AI and the validity of AI, and the patients, maybe some of them are critical but . . . there’s also the opinion “ah it’s an algorithm, it’s smart, it was built by Google or DeepMind or IBM or whoever so it must be right”’ (Interviewee 6). On the other hand, and drawing this time on dystopian media portrayals of AI, some interviewees, particularly in the United Kingdom, perceived threat discourses compounded by ‘bad news stories’ (Interviewee 4) to be factored into health professionals fear of using any AI-enabled technology. Particularly, with reference to such dystopian portrayals of AI, many UK interviewees in particular emphasised the need to close the ‘knowledge gap’ (Interviewee 9) through better science communication (‘we need to engage with the public more so they understand that allowing people to use this data does actually provide benefit’ (Interviewee 4)). Interviewee 2 explained,

what I have found is a misunderstanding of what AI can do . . . and then when you sit down . . . and you explain the process, and you explain actually how distorted the media gets when they get concerned about AI . . . in a way that they understand . . .. it’s usually breaking it down into more lay terms and giving case study examples . . ..

The majority of interviewees described the ‘huge push’ to close the knowledge gap, which was currently underway to ‘*gain public trust*’ in AI (Interviewee 4). However some interviewees (*n* = 3) were less hopeful of the benefits of this approach; Interviewee 12 explained that health professionals may not want to understand the uncertainty attached to AI algorithms. Exemplifying their point using blood tests, this interviewee raised questions about the effectiveness of AI explainability strategies to counter AI-associated hype:

the answer we got back from doctors, when we actually tried to show them and communicate this uncertainty, was ‘why would you show me something you’re not sure about? It’s not my job necessarily, to make that adjudication . . .’ So part of that is, like, when you get a blood test, it just tells you what your measurements are. It doesn’t give you any uncertainty around it. And the decisions about whether you do something is often very binary . . . And doctors are really used to thinking that way . . . and so . . . introducing that uncertainty with the people I’ve worked with, they don’t want to see it. A handful of people do and want to dig deeper into the results . . . most people were like, ‘I have a minute to process this information and make a decision’, . . ..

Finally, a remark made by one interviewee, Interviewee 15, pushes back on the idea that closing the knowledge gap is a silver bullet to addressing hype, and reflects an earlier point made about the inherent complexity, and lack of understanding, of the AI field, even by many AI researchers themselves. This interviewee, who spoke soberly about their own AI research, seemed still to be influenced by AI hype, remarking with relation to another AI field, ‘I really do think of it [AI] as a potential weapon’. This reminds us that even if researchers work in the broad areas of AI and have clear understandings of the technology, they do not need to be far removed from their area of specific expertise to be influenced by sensationalised public depictions of AI, making the strategy to close the ‘knowledge gap’ more difficult. This point also raises another question with regard to where interviewees draw their ideas about utopian and dystopian public portrayals of AI systems, and how their perceptions align with the actual portrayals.

### Newspaper reporting of AI in the context of health

In our analysis of newspaper articles reporting AI health research and care, the majority of articles (*n* = 78, 86.7%) portrayed AI systems as a positive, hopeful development, with *n* = 8 (8.9%) and *n* = 4 (4.4%) articles portraying them in a balanced/neutral or negative manner, respectively. The focus of the news articles is shown in Table 1. Our newspaper sample consisted mainly of publications from the United Kingdom (*n* = 79, 87.8 %). In the United Kingdom, *The Times* (*n* = 15), *Daily Telegraph* (*n* = 14) and *Guardian* (*n* = 10) published the most articles; in Canada, the *Toronto Star* (*n* = 4/11) had the highest prevalence of articles.

**Table 1.** Main focus or topic of news articles, and how topics are portrayed.

Focus or topic of news article	Number of articles with positive/hopeful portrayals	Number of articles with a balanced portrayal	Number of articles with a negative portrayal	Total number of articles (%)
Current/future uses of AI systems: clinical diagnosis and treatment	29	4	1	34 (37.8)
Current/future uses of AI systems: clinical prediction	17	0	0	17 (18.9)
App, chatbot, other population health application	9	1	2	12 (13.3)
Current/future uses of AI systems: hospital management	8	2	0	10 (11.1)
News: AI system used in pharmaceutical Research and Development	7	1	1	9 (10.0)
Other (e.g. AI using patient data; AI-assistive technologies)	8	0	0	8 (8.9)
Total	78 (86.7%)	8 (8.9%)	4 (4.4%)	90

AI: artificial intelligence.

When AI systems were portrayed in a positive light, they were described as a unique and exciting solution to contemporary health issues – a ‘breakthrough’, which was ‘transformative’, ‘revolutionary’ and ‘life-saving’. Further strengthening this narrative, present affirmative or future tenses without a qualifying hedging adverb (such as ‘possibly’), or the use of the conditional tense (‘could be [. . .]’), were used in *n* = 50/90 articles to portray AI as an already established and trustworthy intervention. For example, the *Daily Mail* was one of a number of newspapers which reported the results of an AI dementia detection system, headlining, ‘Hi-tech computers detect dementia years in advance’ (*Daily Mail* yet, a scan of these news articles revealed most provided little to no discussion about the study’s small sample size or preliminary findings. Those articles that did, only mentioned these facts at the end. Another example was the headline, ‘Cancer treatment revolution: AI helps doctors outwit disease’ (*i-Independent Print Ltd*). The claim of both a ‘revolution’ and an aid *ready* for doctors sat in contrast to the fact that it is not available for clinical use at the time of writing – a point noted much further down the article.

Even when conditional tenses and hedging adjectives were used, AI was framed in forward-looking language that urges the reader to consider the transformative potential of AI, and in some instances, even warning of the dire consequences should AI not be ‘embraced’ (*Sunday Telegraph*). An article discussing Amazon’s Alexa speakers headlined, ‘Smart speakers could diagnose your sickness inside a decade’ (*The Times*) elaborated on exciting future diagnostic applications of the technology, despite this being very early-stage research. Similarly, another news article headlined ‘Facebook could be a tool to spot early signs of depression’ (*The Daily Telegraph*), focused on the future potential of AI. This article was particularly interesting: the AI system being reported analyses the language of Facebook users to detect signs of depression, yet no mention was made to the myriad of associated ethical, social and practical challenges, such as analysing Facebook content without the consent of the Facebook user, the difficulties with identifying depressive symptoms (and what this could mean in a clinical/health context), and issues with notifying users and offering (or failing to offer) adequate support and/or treatment.

Finally, news articles contrasted how they depicted AI agency. On one hand, a number of articles underlined the performance ability of AI to be ‘as good as experts’, and in some cases, surpassing human ability (*n* = 10), with headlines reading, for example, ‘Computers have outperformed doctors in diagnosing neurological illnesses and retinal disease’ (*The Times*). In contrast, more prevalent, and reflecting our interviewees’ perceptions, AI was presented as systems eventually becoming benign and useful aids to healthcare staff and systems (*n* = 37). For instance, ‘Science using A.I. as guide to cancer’ (*The Sun*), and ‘Technologies including artificial intelligence and robotics can help the NHS become more efficient’ (*The Sunday Telegraph*).

## 4. Discussion

As has been shown in similar studies (Laï et al., 2020), our interviewees viewed AI systems solely as a methodological instrument, one of a number of research tools, non-exceptionalist, not exciting, and uncertain in terms of its capabilities. They imagined the field of implementing AI-based solutions as far from reality, and that predictions are ‘goals that motivate and drive their work, not accurate depictions of the state of the art ..[..].. as with any nascent and emerging field, what is behind the curtain is full of contested boundaries and uncertainties, methodological challenges and epistemological pitfalls’ (Elish and Boyd, 2018: 69). Our researchers juxtaposed these views of AI systems with their perceptions of how AI systems are publicly constructed as either over-enthusiastic in terms of their benefits, or as posing an imminent danger. While these perceptions are supported by the literature (Elish and Boyd, 2018), they were only partially supported by our news article analysis. While several articles reported on dystopian eliciting stories associated with AI surpassing health practitioners’ abilities, this (almost) dystopian narrative was of much less focus than our interviewees suggested in their narratives. Our news articles overwhelmingly constructed AI health research using the socio-technical imaginary (Jasanoff and Kim, 2009) of optimism, newness and certainty. As such, interviewees were likely drawing their perceptions about the public portrayal of AI from the reporting of AI more generally rather than the reporting of health research specifically. This is important to note, since if AI researchers are unable to see the potential impact of their work through the public’s eyes (or have misconceptions about this), this could have implications for the way in which they frame their own research when communicating about it to both professional and public stakeholders.

The separation of sober representations and uncertainty of AI methods by interviewees against the more sensationalist expectations of the media is unsurprising given the literature on ‘alien science’, uncertainty and the generation of expectations about science (Brown and Michael, 2010; Collins, 1999; Hedgecoe, 2006). Another way of interpreting this separation, and a way that also reflects the non-exceptionalist properties our interviewees gave to AI systems, is that our interviewees’ closeness to their work normalises them to their AI research methods and tools. What at a distance is perceived as exciting and new, close up, with the monotony of daily use, is something less exciting, more ‘normal’ and therefore non-exceptional.

Our interviewees’ focus on the uncertainty of their work is in line with Pickersgill’s (2016) concept of epistemic modesty, in which researchers admit the uncertainty, ambiguity and opacity of their field of study. The role for describing their work as uncertain seemed to relate to the knowledge machinery of scientific practice, whereby interviewees’ discussed problems associated with their work just like when they are talking informally to colleagues (Pickersgill, 2016). It is likely that such discussions of uncertainty were also used by interviewees to dampen the expectations around AI systems, whereby interviewees were acting responsibly by communicating with scientific professionalism and rendering problematic the research of competitors (Pickersgill, 2016). This, explains Pickersgill (2016), is associated with an ‘*empathetic citizenship*’ (p. 197), whereby researchers have a long-standing, intrinsic desire to try and ensure that false hopes and expectations on the part of non-scientists are lamented and, where possible, minimised (p. 197). In fact, interviewees drew on the ‘them and us’ narrative to situate their responsible selves at a distance from other researchers who they perceived as being inappropriately ‘epistemically ostentatiousness’ (Pickersgill, 2016) – framing AI research as new, innovative, ‘cool’ or exciting for the self-interested purposes of attracting investment.

Finally, we cannot conclude from the above that our interviewees enacted these responsibilities themselves, or that they did not adopt similar epistemic ostentatiousness outside of the interview setting. It has long been known that researchers describe their research using a number of context specific discourses, reporting it to be more or less uncertain depending on the specific circumstance (Evans et al., 2009; Gilbert and Mulkay, 1984). In fact, researchers must often learn to manage sensationalist expectations (Gardner et al., 2015) by navigating the regimes of hope in their own work (epistemic ostentation) with regimes of truth (Moreira and Palladino, 2005; epistemic modesty). However, our interviewees’ narratives hinted at the authenticity of their self-proclaimed responsibilities and their desire to maintain this epistemic modesty as much as possible. Interviewees seemed to be concerned about communicating their AI research to health professionals appropriately, and seemed to ‘care about the production of knowledge, about the enthusiasm that this can stimulate in non-scientists, and about the importance of precision in communicating developments . . . to those without sufficient expertise themselves to adjudicate new claims’ (Pickersgill, 2016: 197). This was described by our interviewees as sometimes being difficult because some health practitioners were perceived to actively *want* to lose the uncertainties surrounding AI systems when using the technologies, preferring the science to remain ‘alien’, just as long as the AI system promised to provide a rapid and efficient decision-making aid. This is problematic for a range of reasons, not least because when ‘alien science’ comes to be seen as ‘simply science’ (Pickersgill, 2016: 194) outside of the research specific environment, decision-making about research funding and use can ultimately be made with little attention paid to the research’s inherent uncertainties.

As a final note, two implications from our findings do not directly relate to this article’s aims – being more related to the initial aim of our research project to explore ethical issues related to AI population health research – but nevertheless, deserve mention. First, our researchers perceived that unrealistic expectations around AI could be addressed by better communication with health professionals/the public, which would improve understanding and build trust. At times, our interviewees’ narratives suggested a deficit-type one-way approach to engagement (researcher to public/stakeholder) to help fill the knowledge gap to build this trust. However, the relationship between communicating AI knowledge and trust in AI is complex. Individuals may not always need (and as we have shown above, *want*) to understand a technology to trust it (and vice versa), and trust in a technology may be more underpinned by trust in broader social systems and networks, such as trust in the institutions developing or implementing the technology, or trust in technology governance more generally, than trust in the technology itself. Trust in a technology may also be related more to experiences, such as individual/organisational experiences of working with technologies and/or beliefs about how technology can be accommodated in their workflows. Also, trust does not always equal trustworthiness.

Second, reflecting on the fact that our researchers seemed to view AI as an unproblematic methodological research tool, social science scholars have long alerted us to the fact that methods and methodological tools are not uncontroversial in that they can be separated from ethics. Rather, methodological tools and design embed ethical choices (Markham, 2006; Markham et al., 2018). It is worth therefore re-iterating, that when developing or using AI software, AI researchers must remain ethically alert to the fact that all research tools, including AI, have biases and assumptions built into them (Berendt et al., 2015; Elish and Boyd, 2018; Gillespie, 2014; Luka and Millette, 2018; McQuillan, 2018; Vis, 2013; Zimmer, 2018).

## 5. Conclusion

This article aimed to explore how HEI population health AI researchers perceive the use of AI systems in their research, and how this compares with their perceptions about the news media portrayal of AI systems in health research. Our interviewees viewed AI systems solely as a methodological instrument, one of a number of non-exceptionalist research tools. This contrasted with the media’s portrayal of AI that optimistically focused on the benefits of these tools. These findings can be understood by the fact that interviewees’ closeness to their work normalises them to their AI research methods, and so, what at a distance seems exciting and new, close up, with the monotony of daily use, seems less exciting. It can also be understood by drawing on Pickersgill’s concept of ‘epistemic modesty’, which, by scientists admitting the uncertainties within their work, plays a role in responsible discussion and dissemination of science research. However we have shown that this can sometimes be hindered by stakeholders to whom the research is disseminated, who may be less interested in hearing about the uncertainties of scientific practice – something that can have implications in terms of the responsible societal use of research, and on the research and policy environment.

## Notes

1
https://github.com/hollobit/COVID-19-AI/
